# Supplementary figures and images for: L-lysine protects C2C12 myotubes and 3T3-L1 adipocytes against high glucose damages and stresses
Source: PLoS One. 2019 Dec 19;14(12):e0225912. doi: 10.1371/journal.pone.0225912 (PMC6922410; doi:10.1371/journal.pone.0225912)

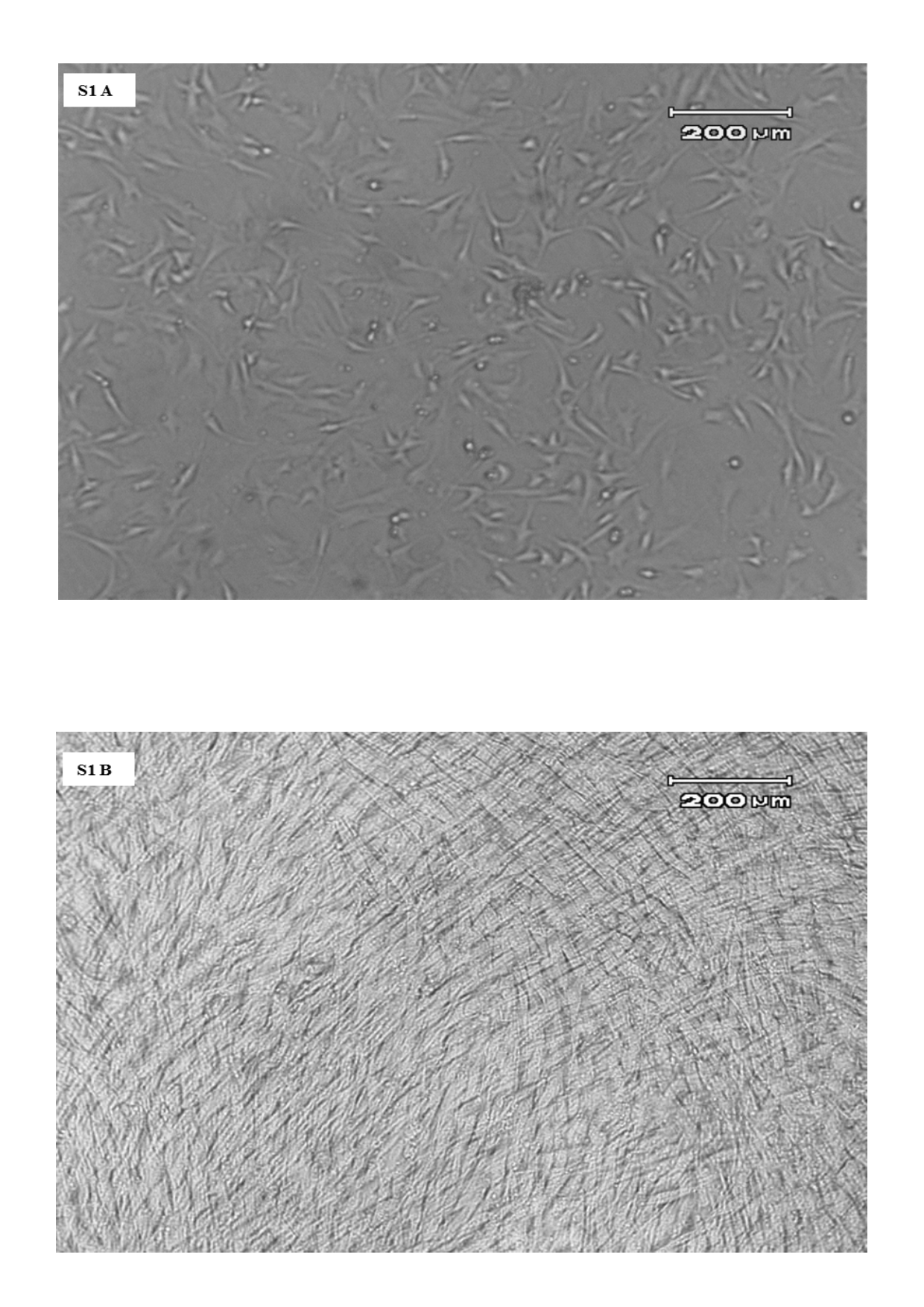

Supplement: S1 Fig — (A) in control medium and (B) in differentiation medium after 6 days. (TIF) [file pone.0225912.s001.tif]

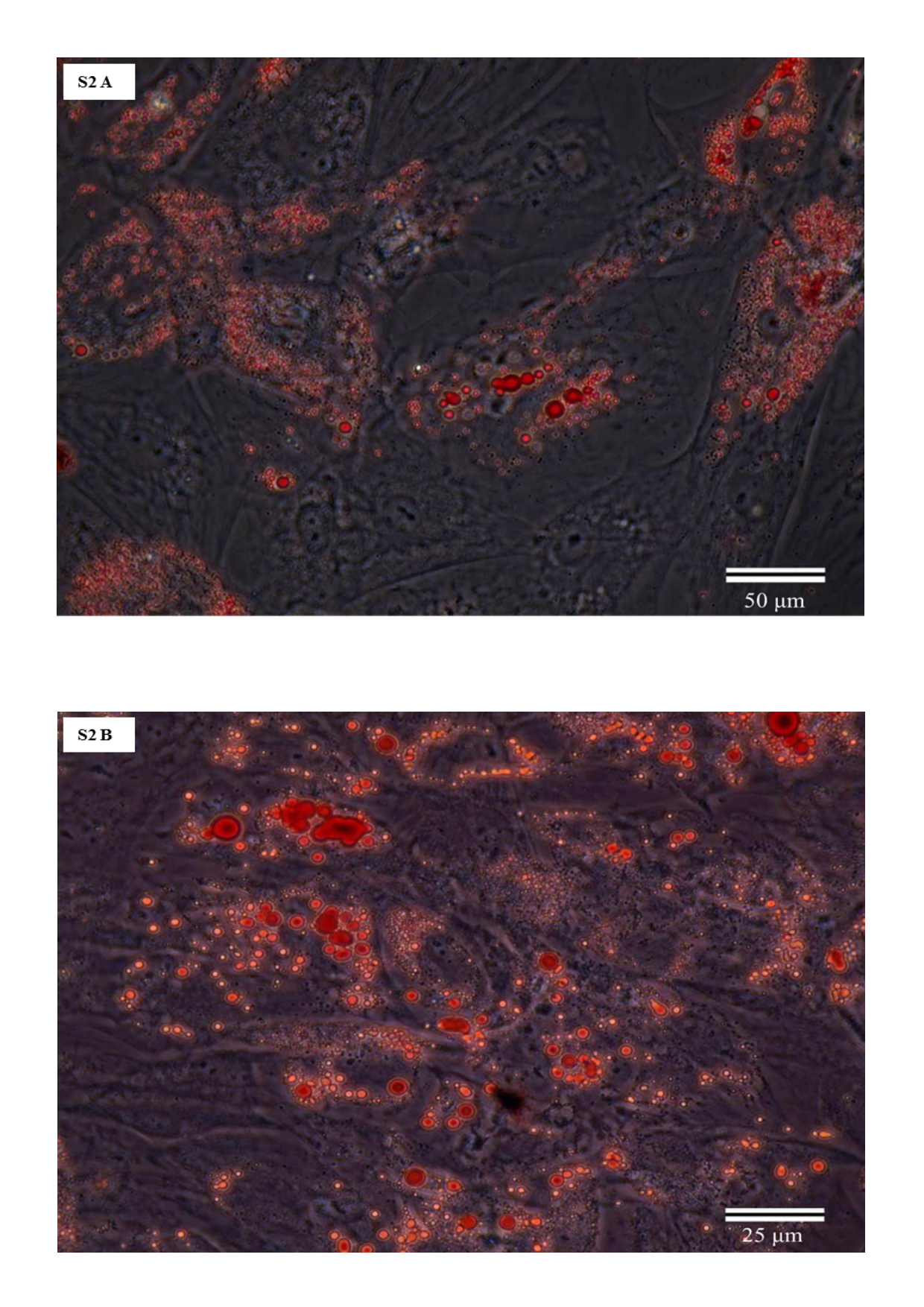

Supplement: S2 Fig — (A) The 3T3-L1 cells staining at first, and (B) after 2 weeks incubation in differentiation media. A significant increase in the lipid droplets stained with Oil Red O, in the peri-nuclear region, indicating the differentiation of 3T3-L1 cells to 3T3-L1 adipocytes. The white marker on the right bottom of the figures indicating a 25 μm scale. (TIF) [file pone.0225912.s002.tif]
