# Supplementary material for: L-lysine protects C2C12 myotubes and 3T3-L1 adipocytes against high glucose damages and stresses
Source: PLoS One. 2019 Dec 19;14(12):e0225912. doi: 10.1371/journal.pone.0225912 (PMC6922410; doi:10.1371/journal.pone.0225912)

Fig 5A

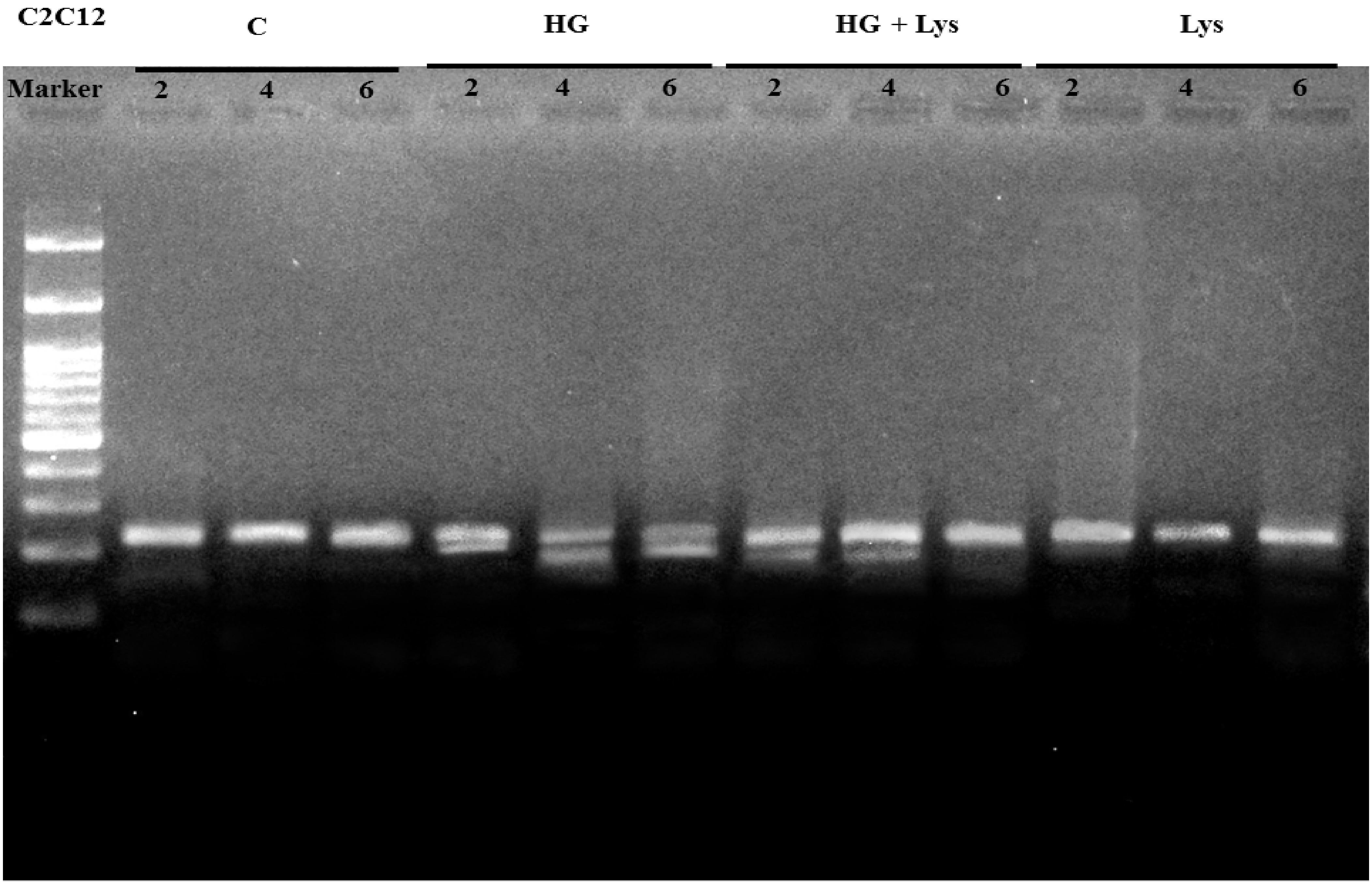

Fig 5B

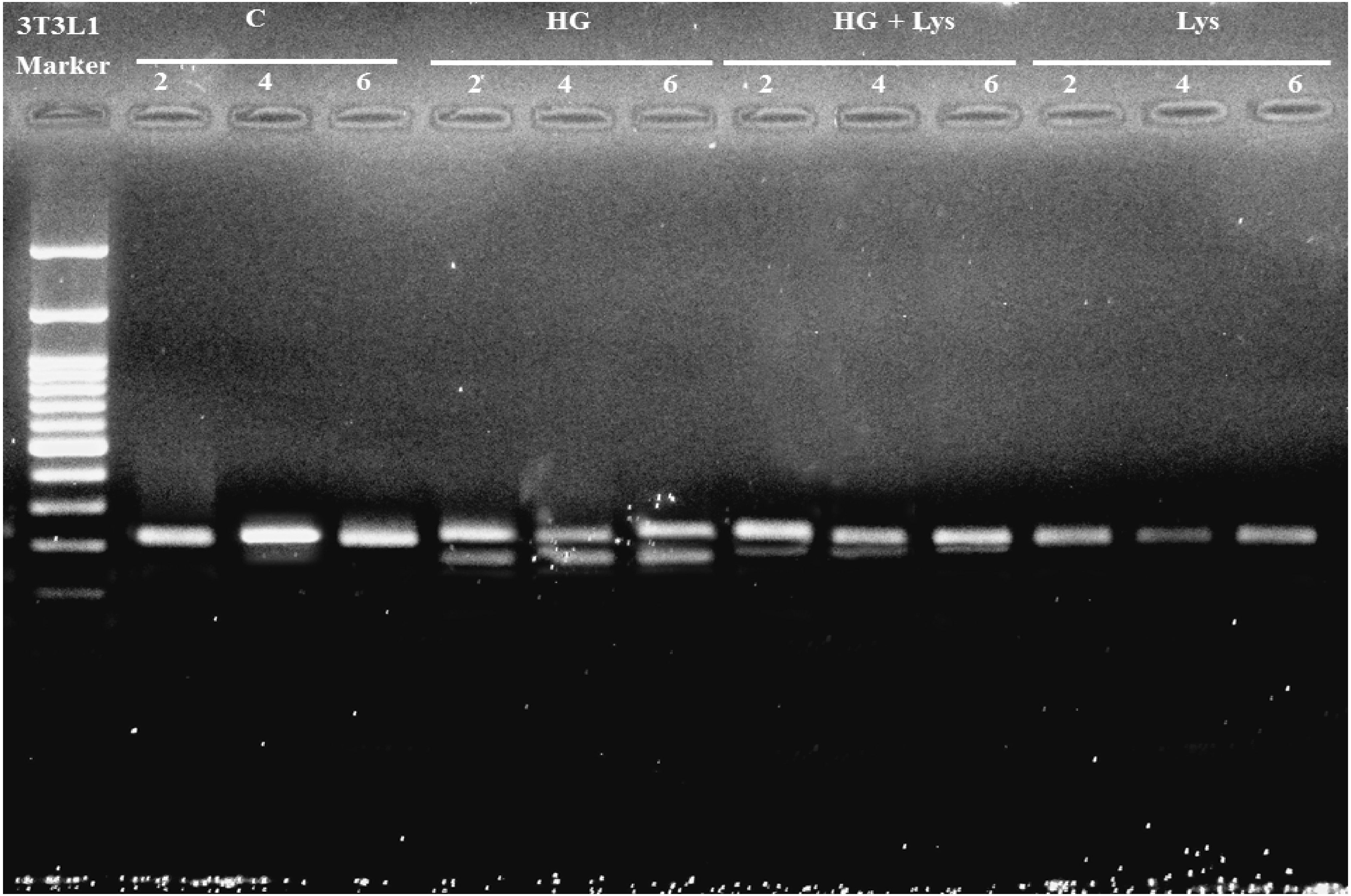

Fig 6A

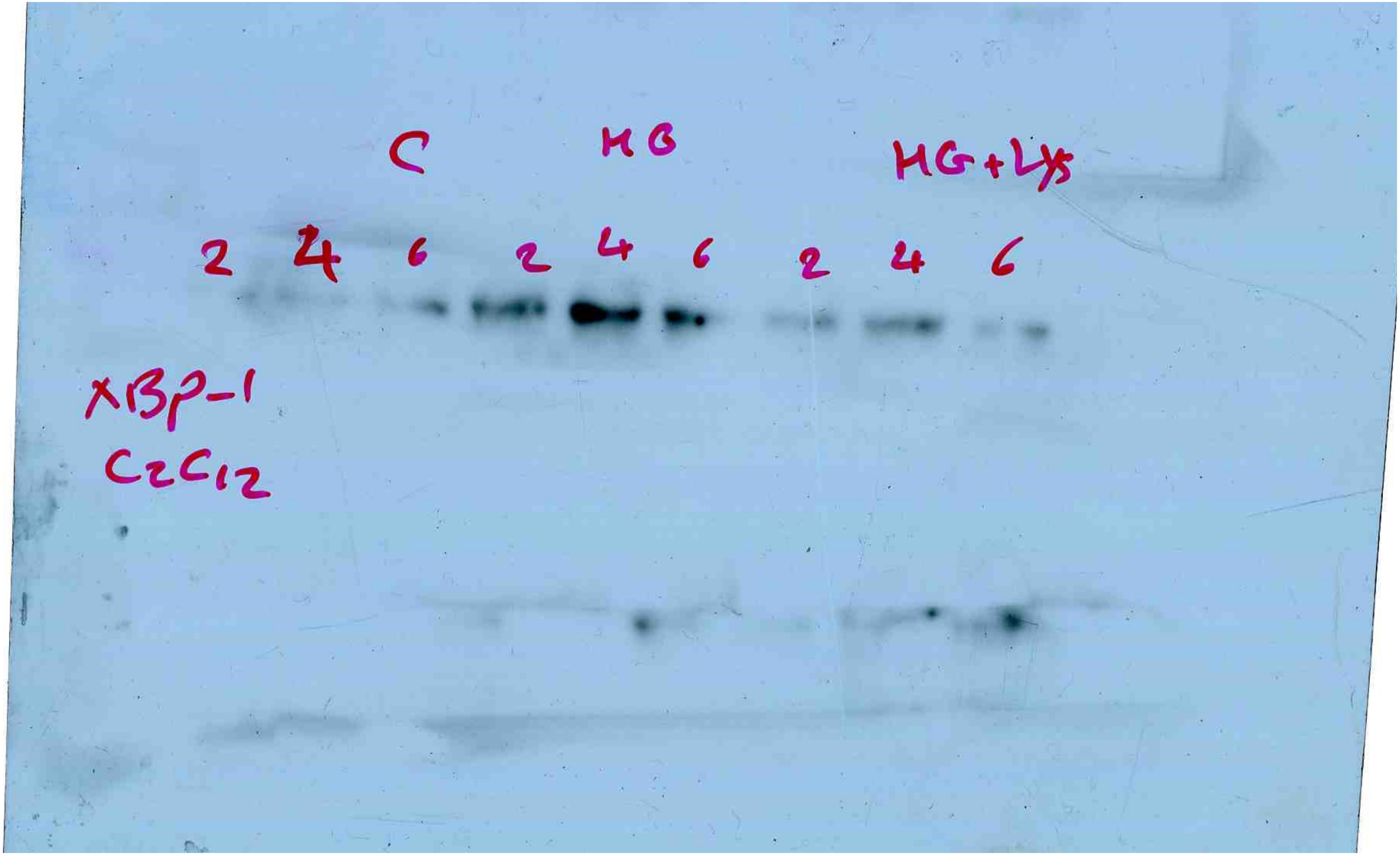

Fig 7A

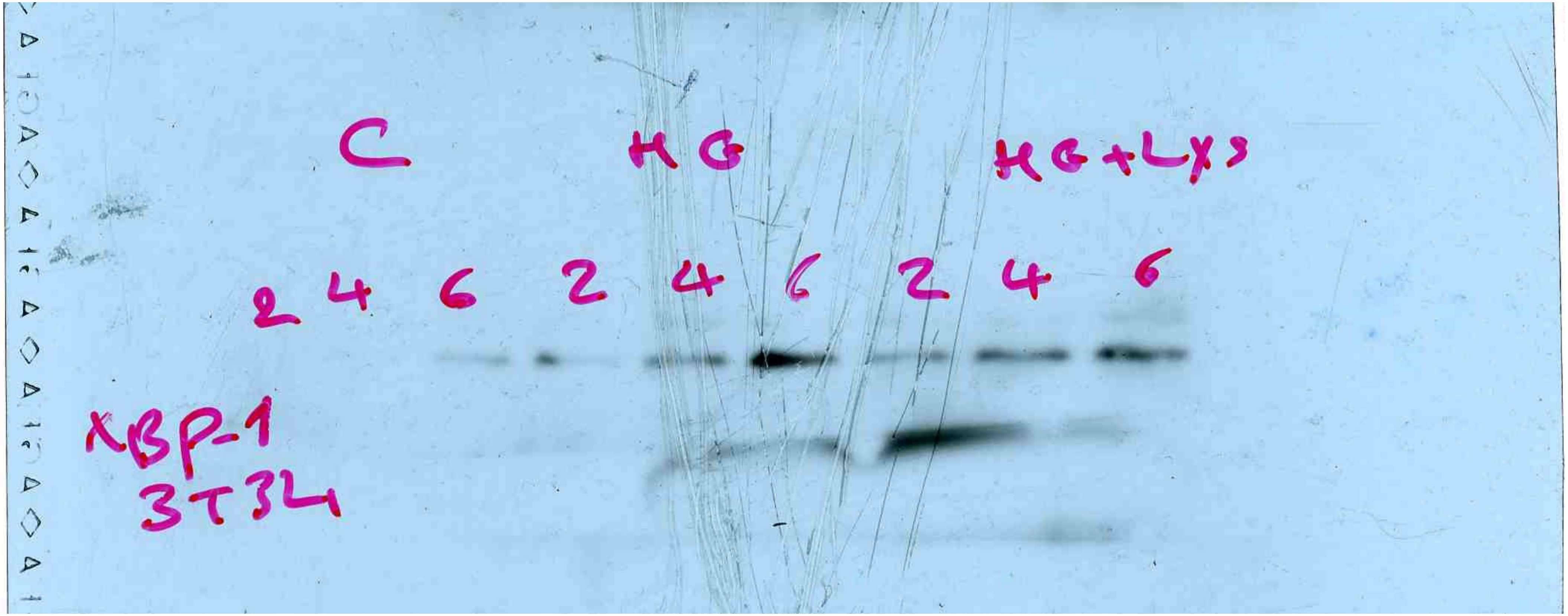

Fig 6A and 7A

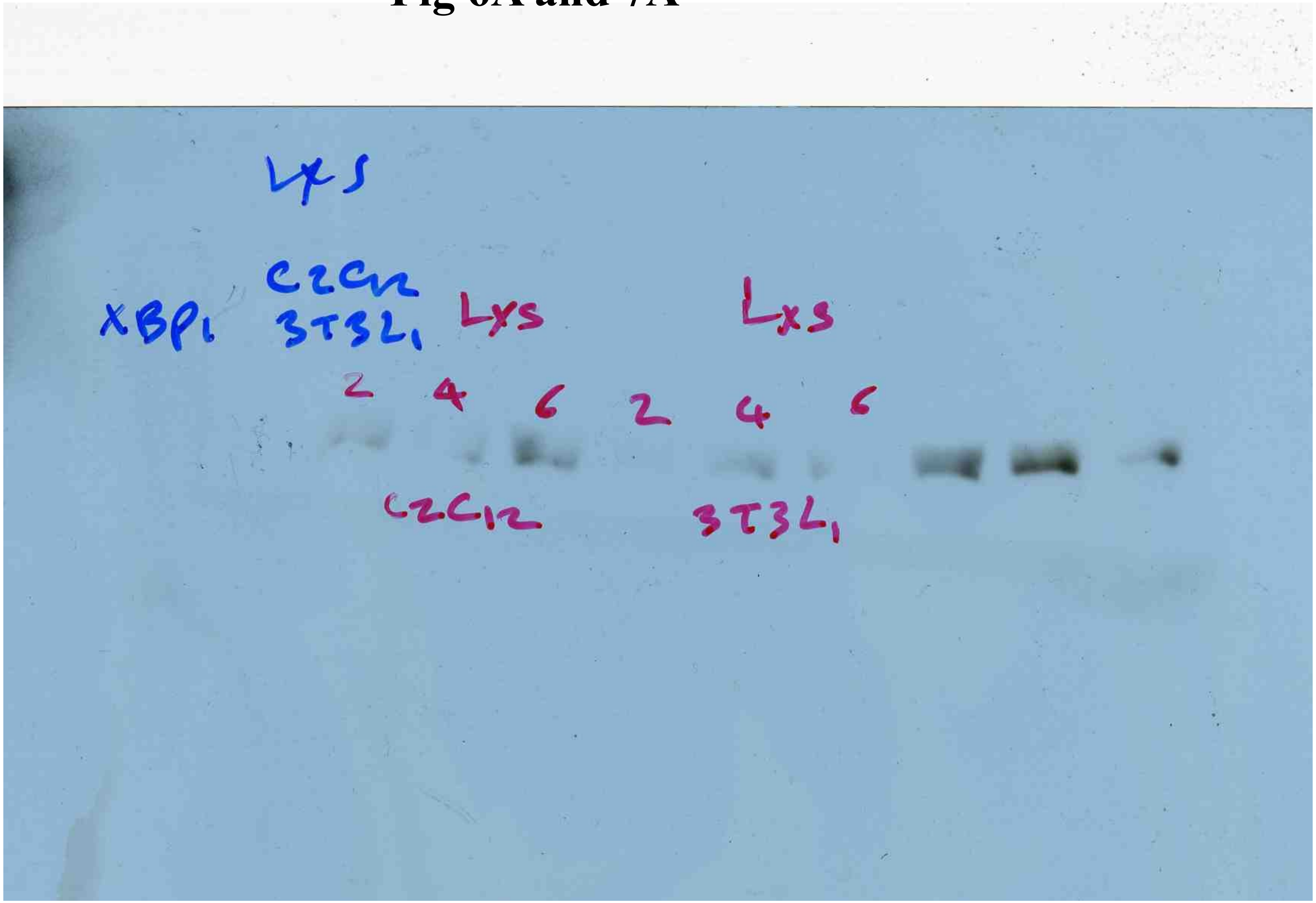

Fig 6A

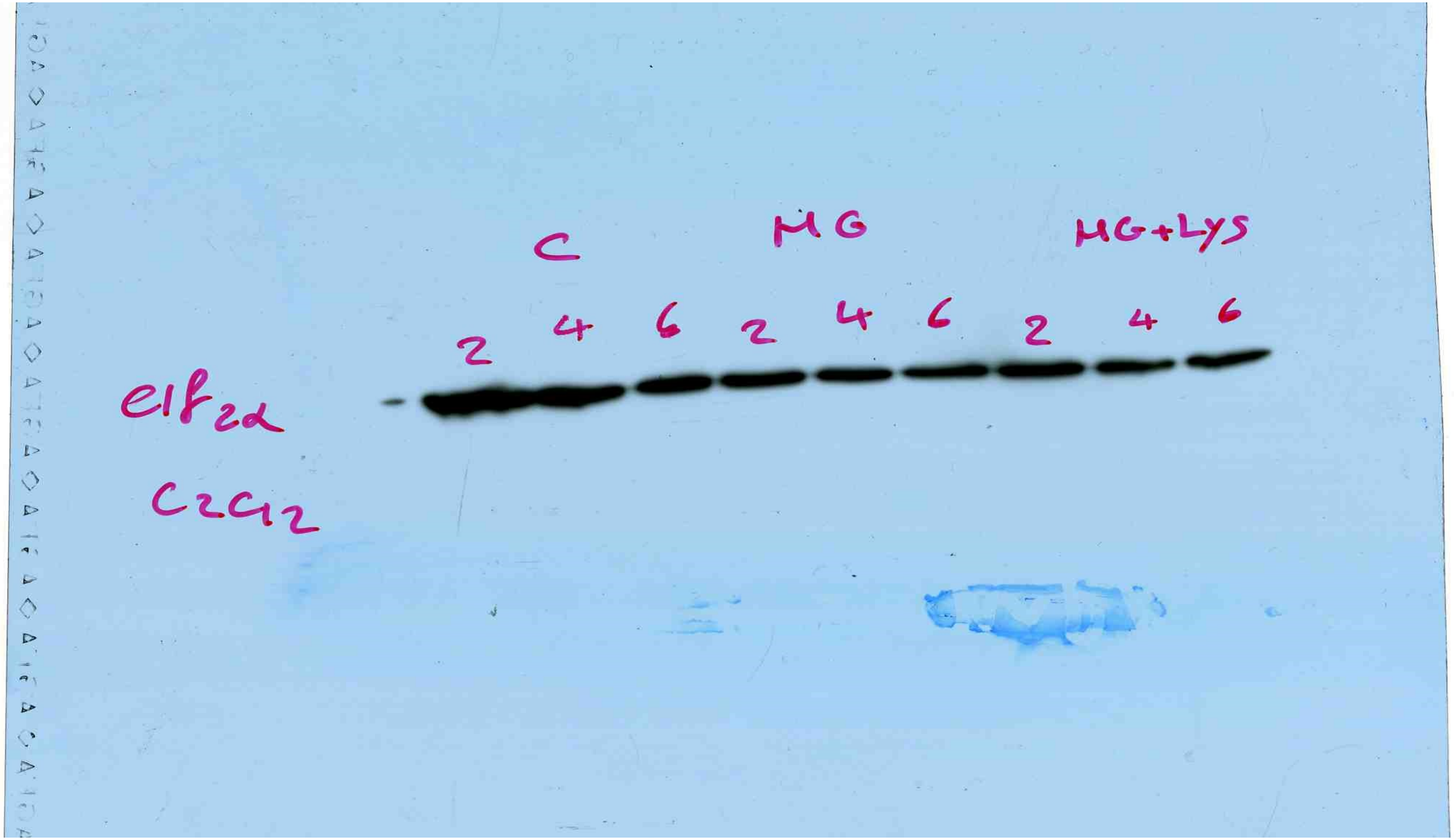

Fig 6A

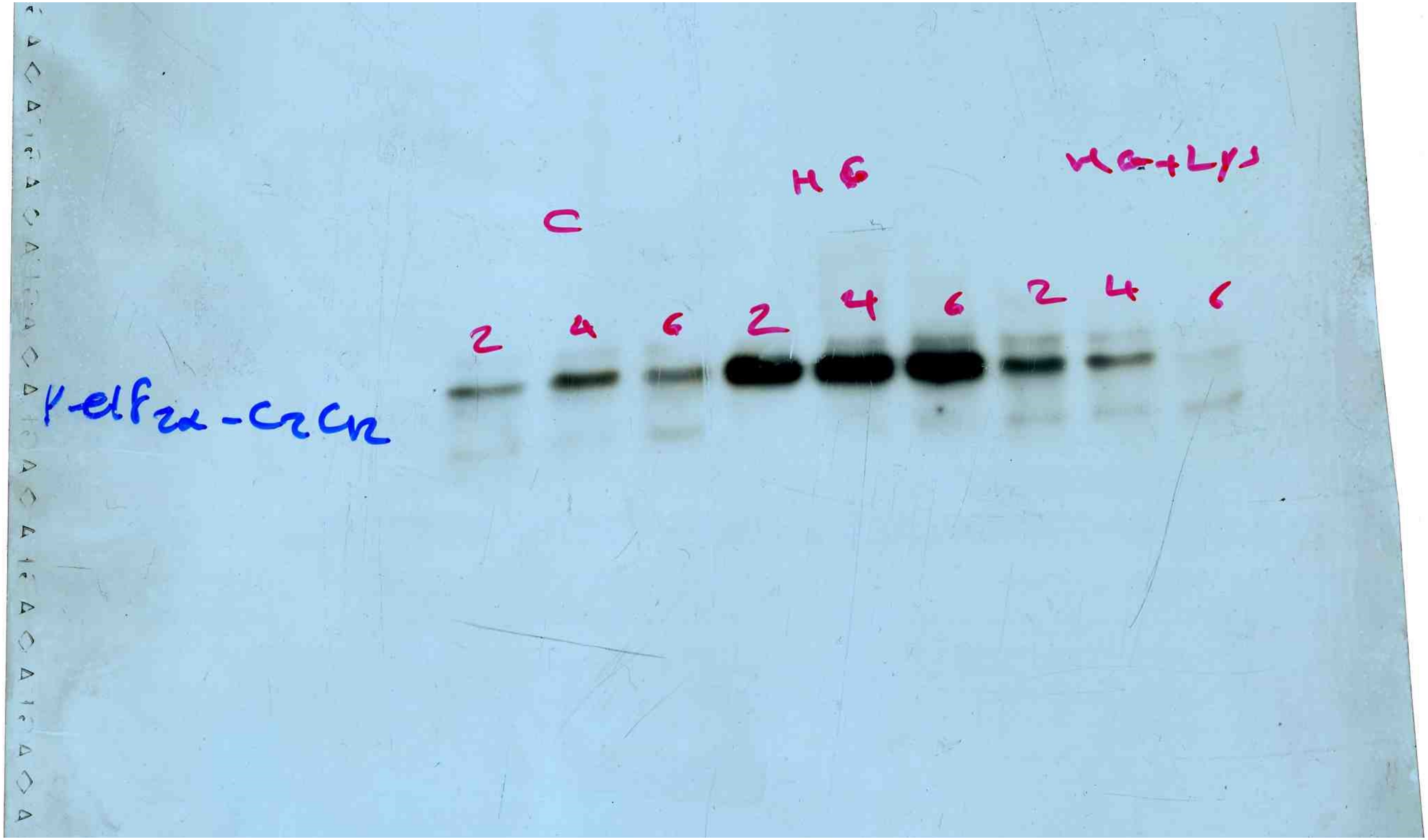

Fig 7A

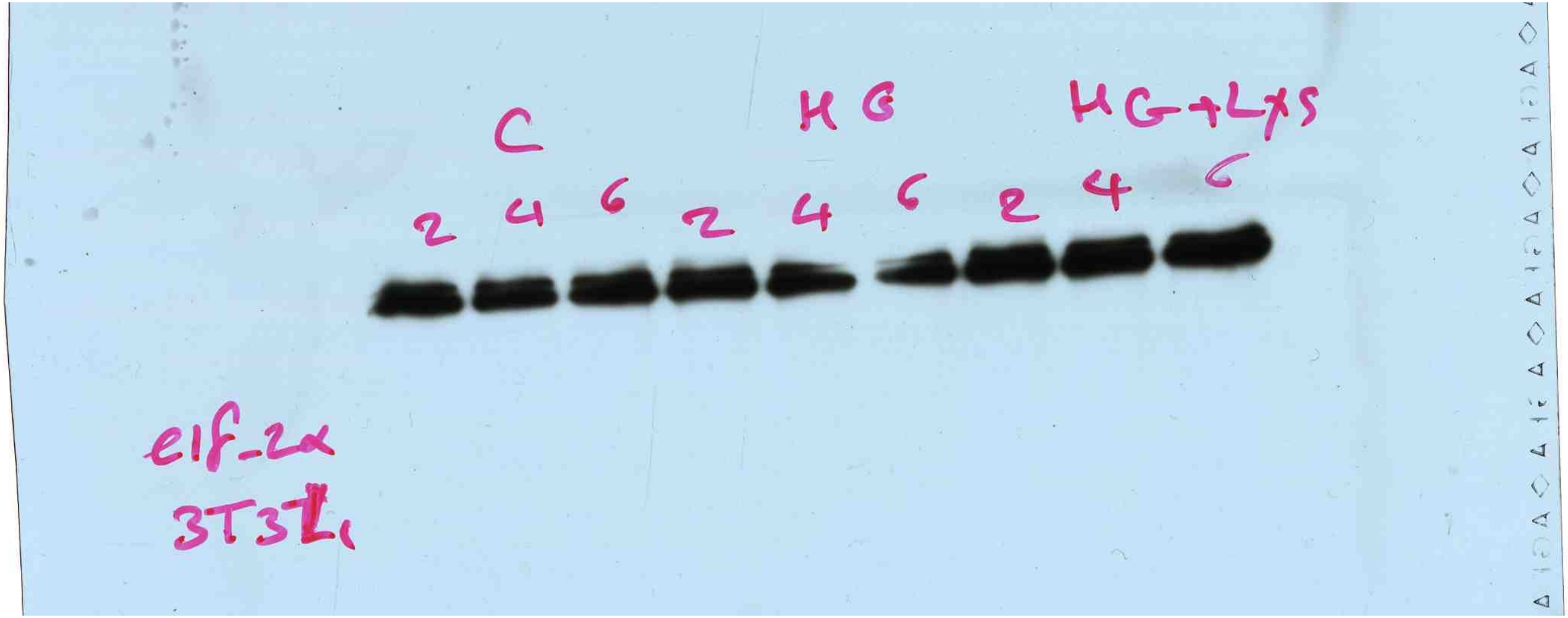

Fig 7A

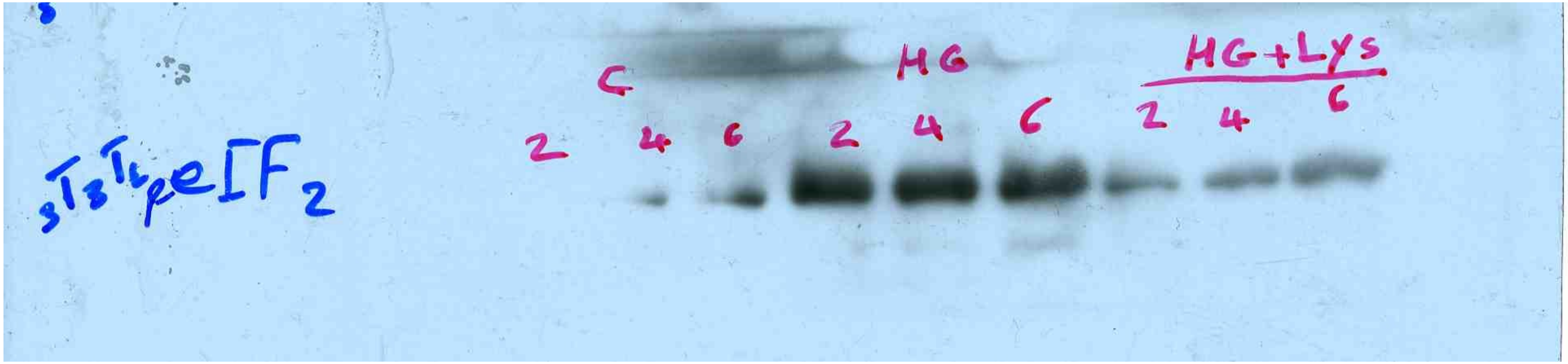

Fig 6A and 7A

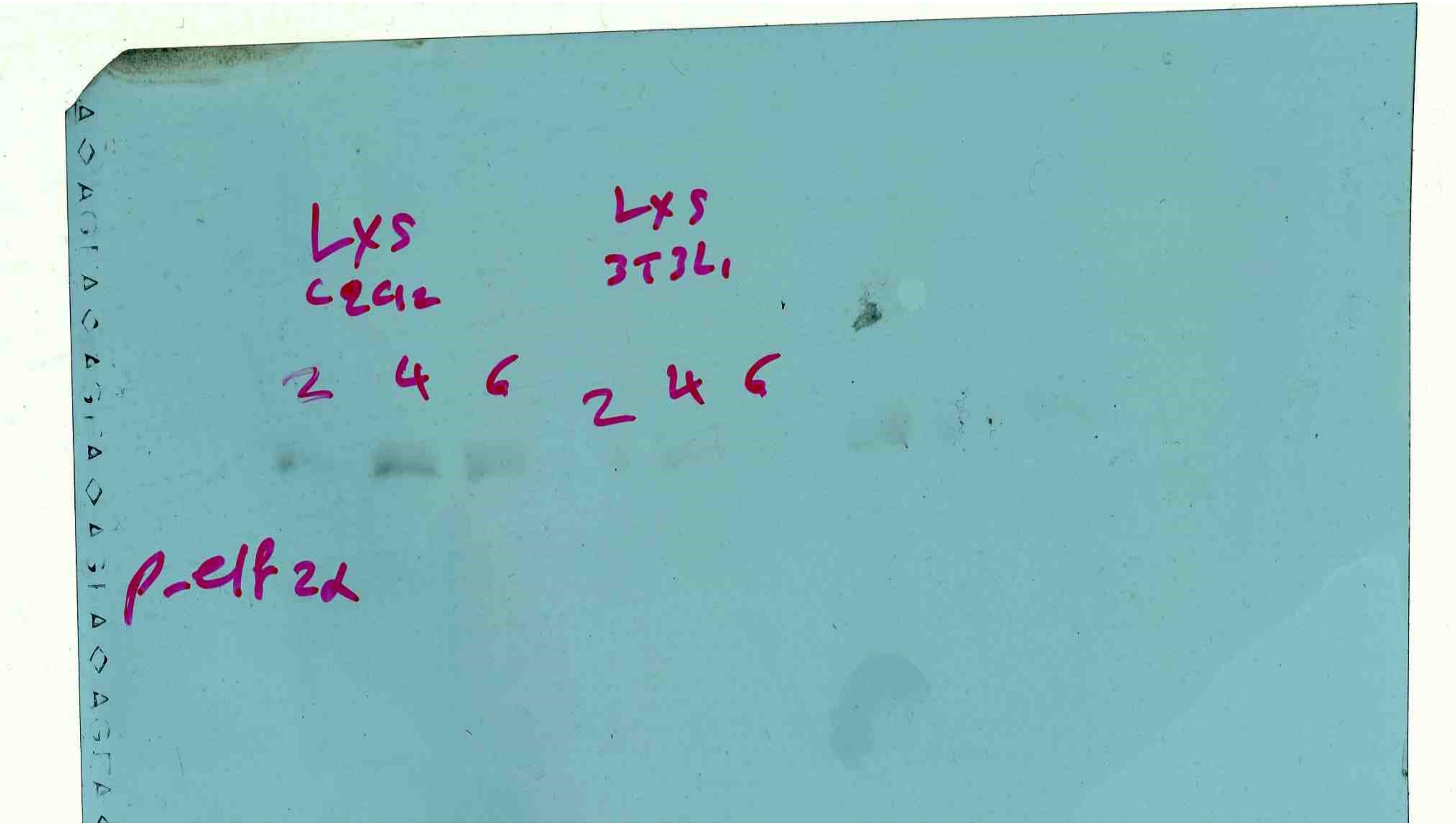

Fig 6A and 7A

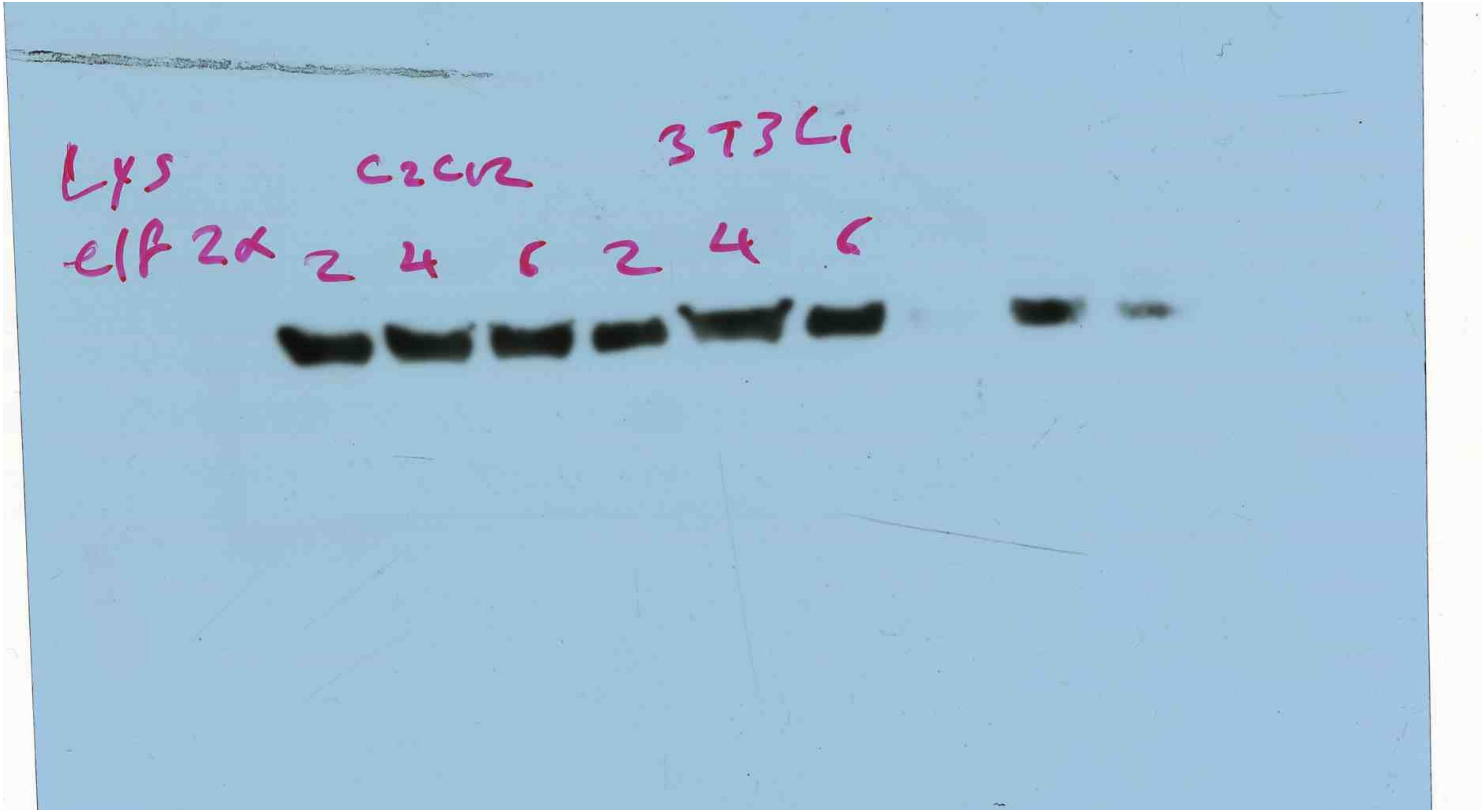

Fig 6A

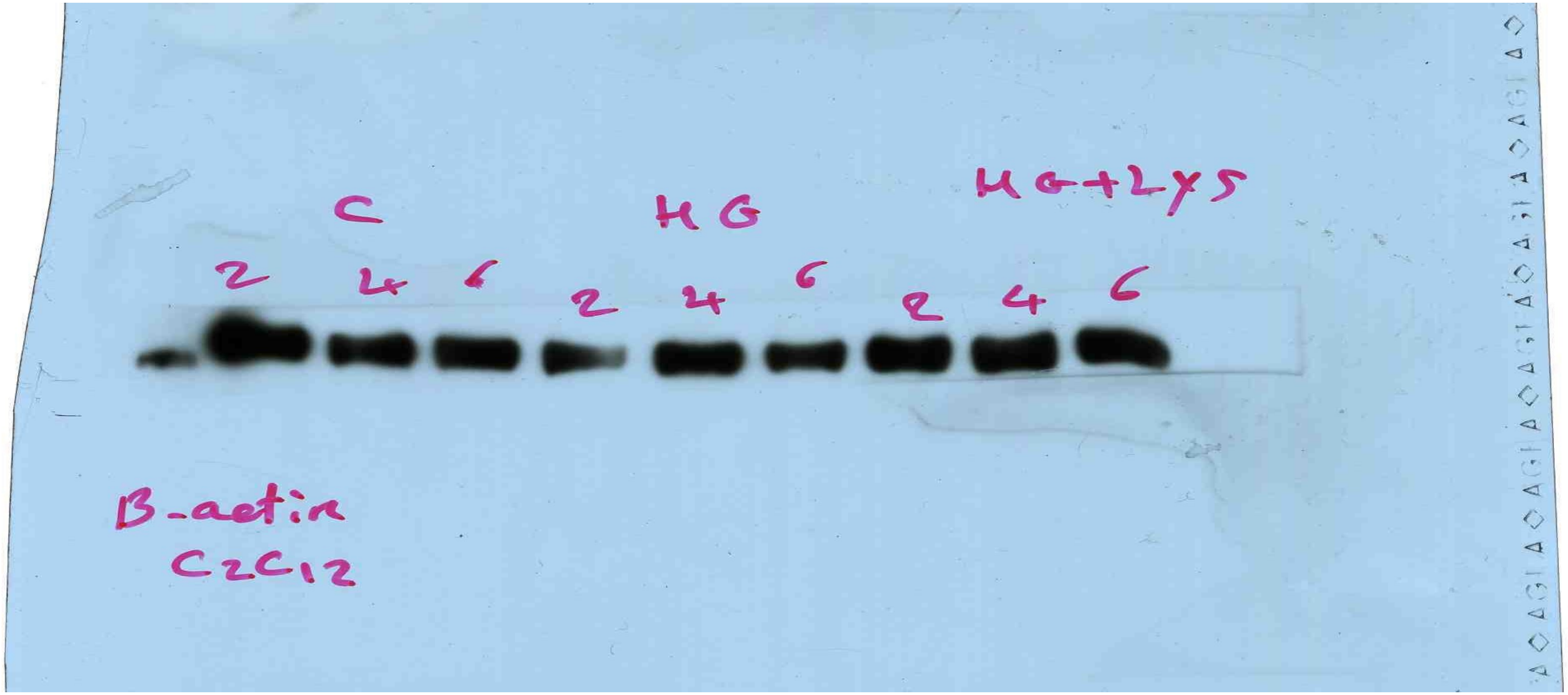

Fig 7A

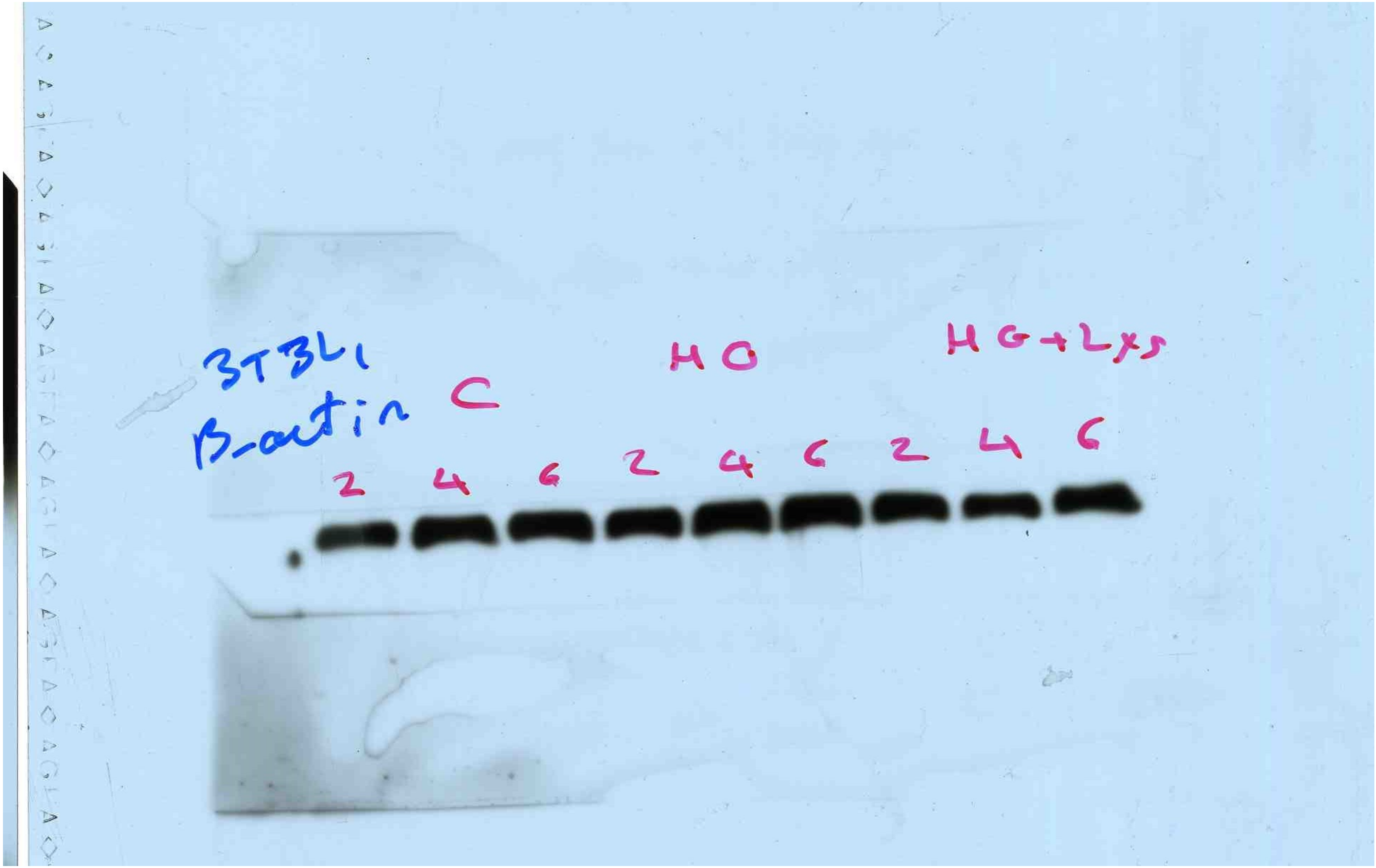

Fig 6A and 7A

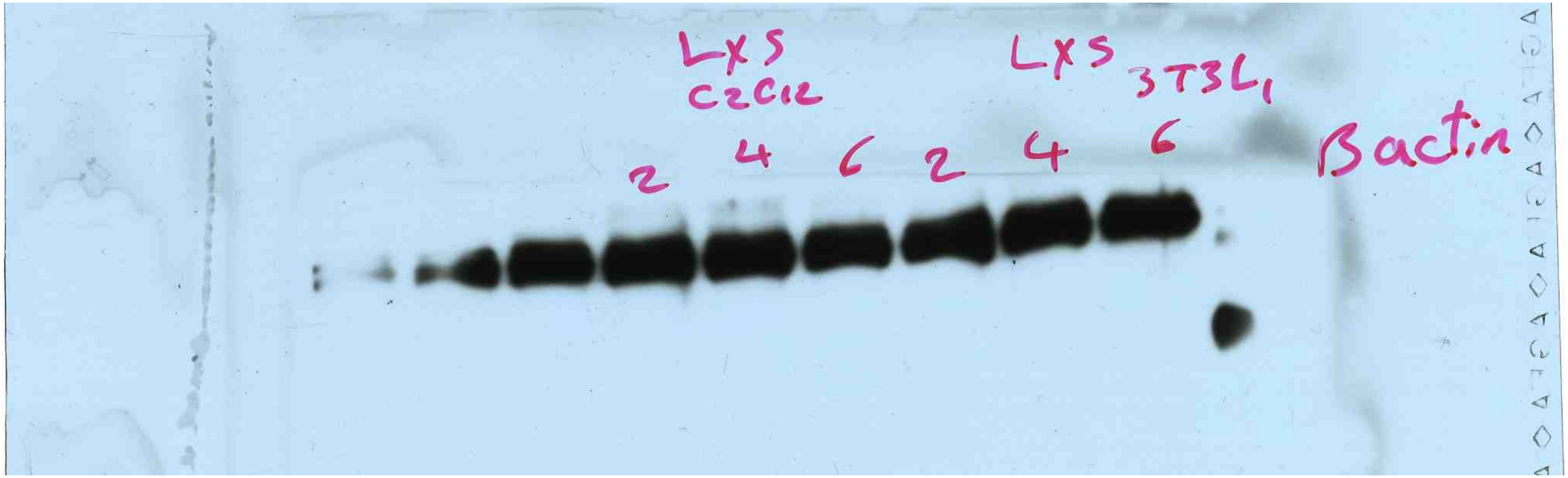

Fig 8A

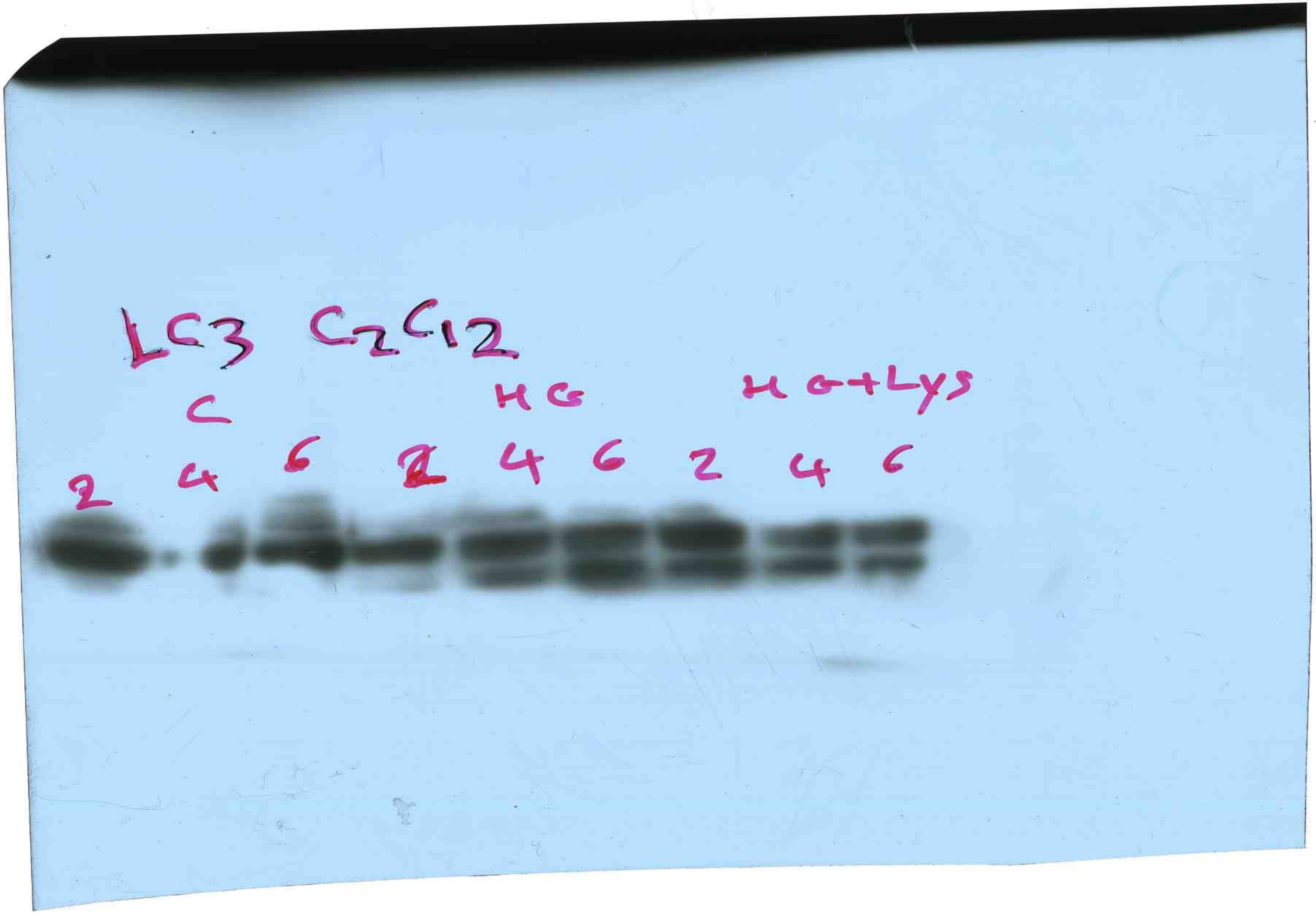

Fig 8A

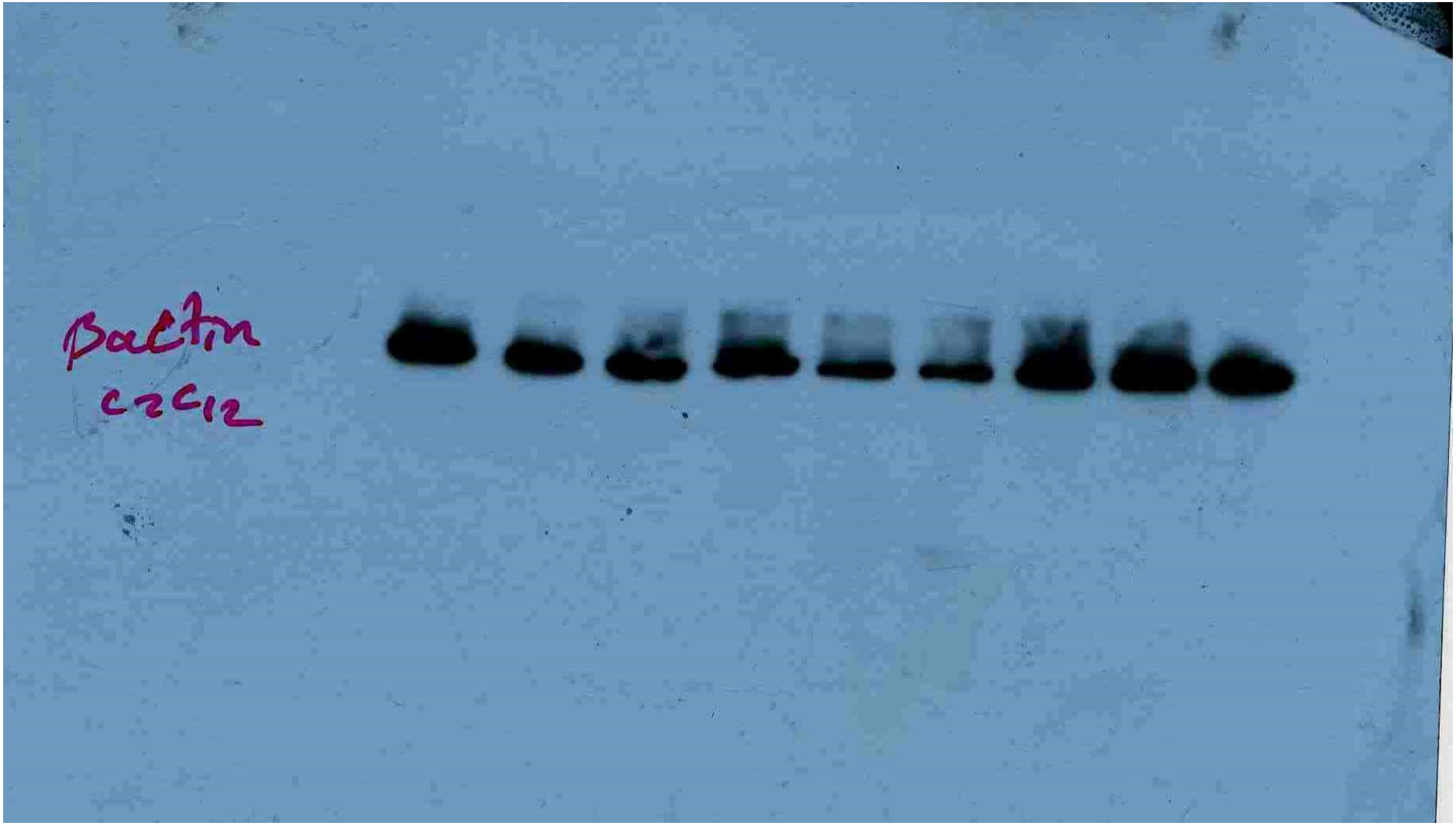

Fig 9A

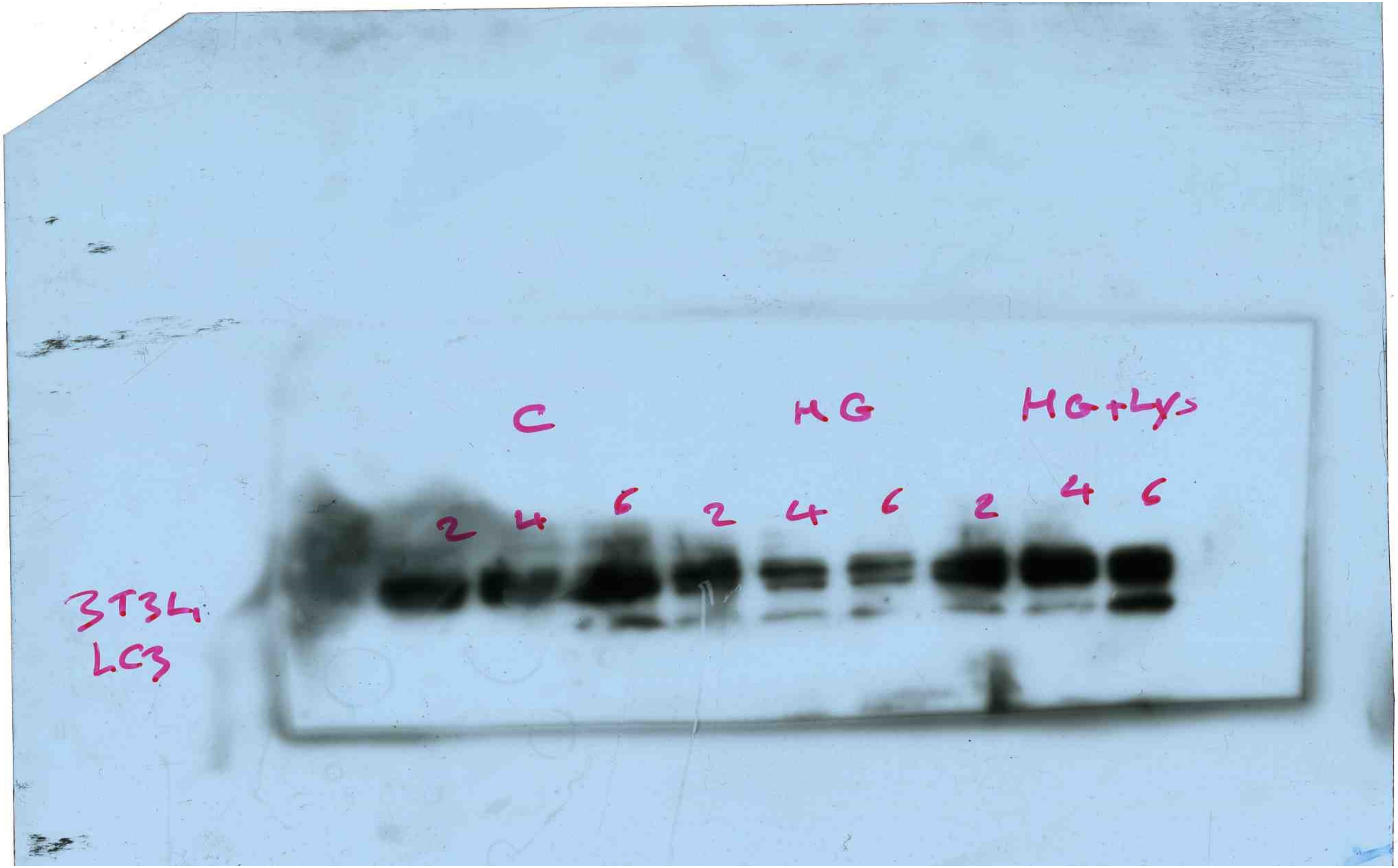

Fig 9A

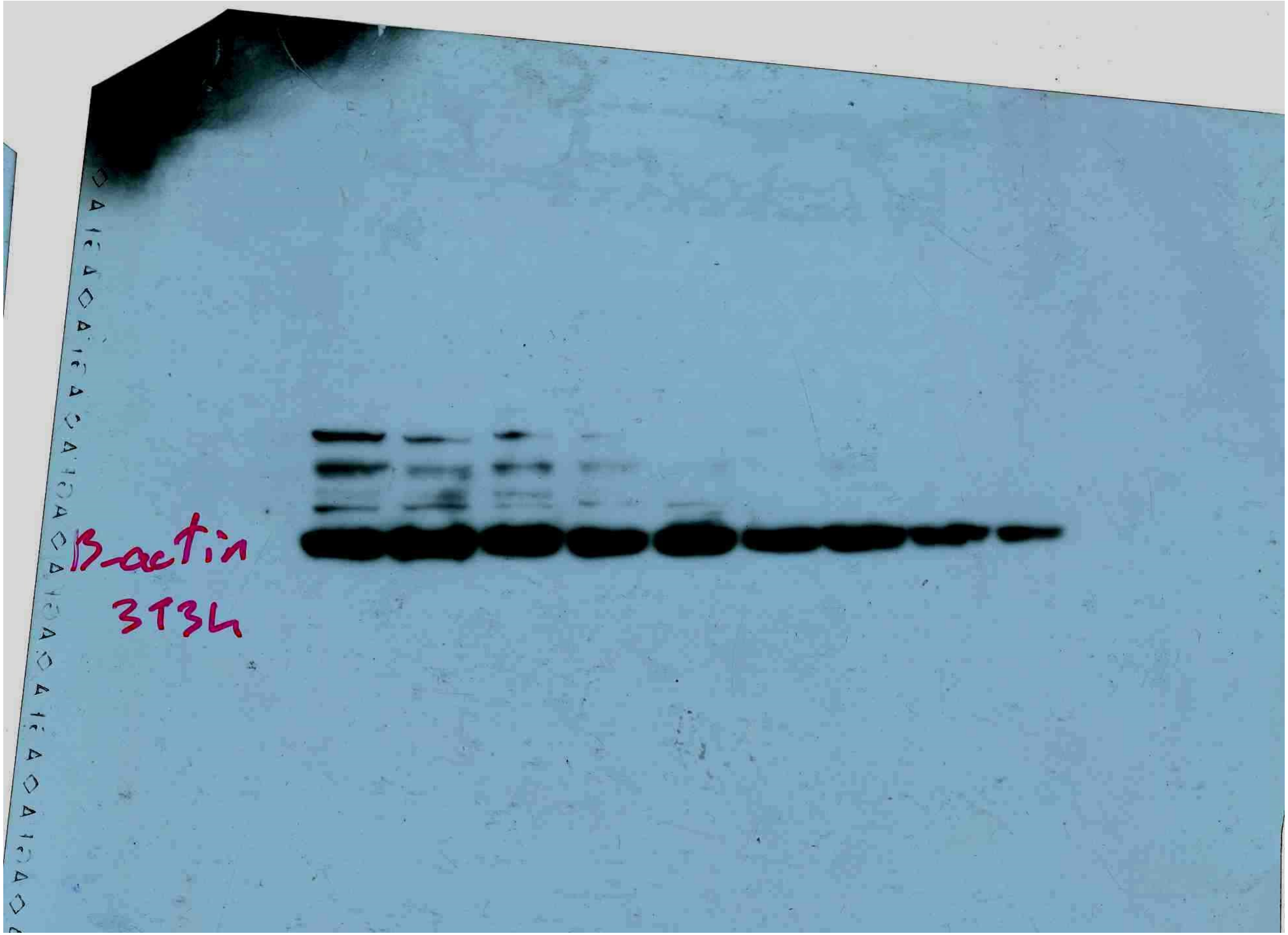

Fig 8A and 9A

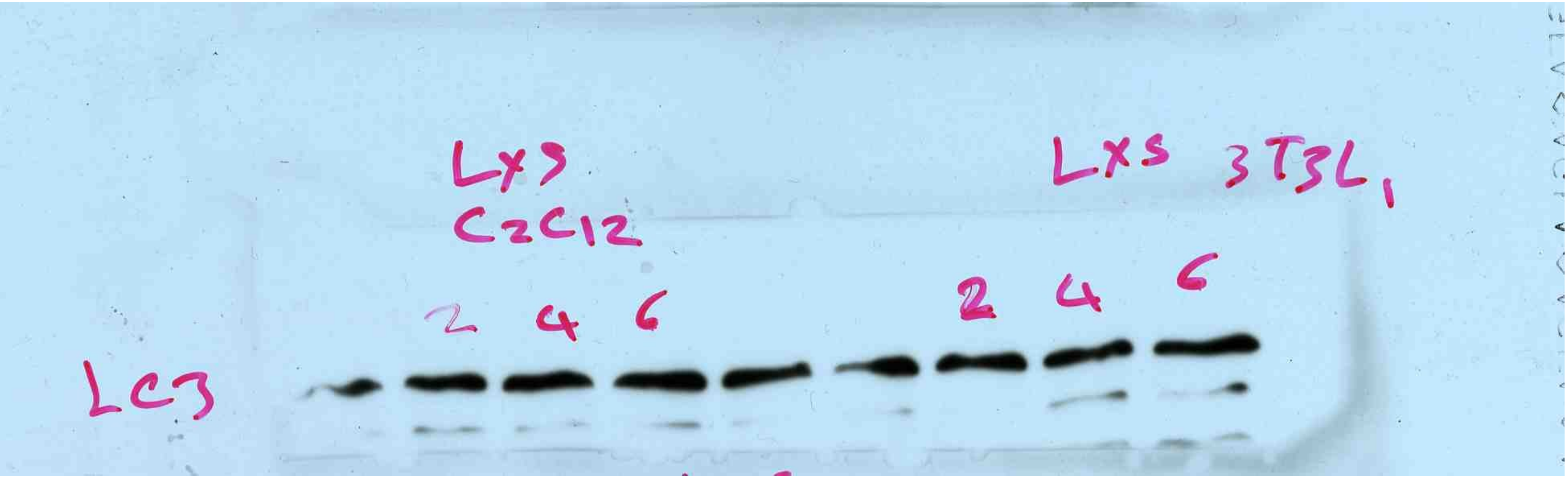

Fig 8A and 9A

B-Actin of 3T3L1

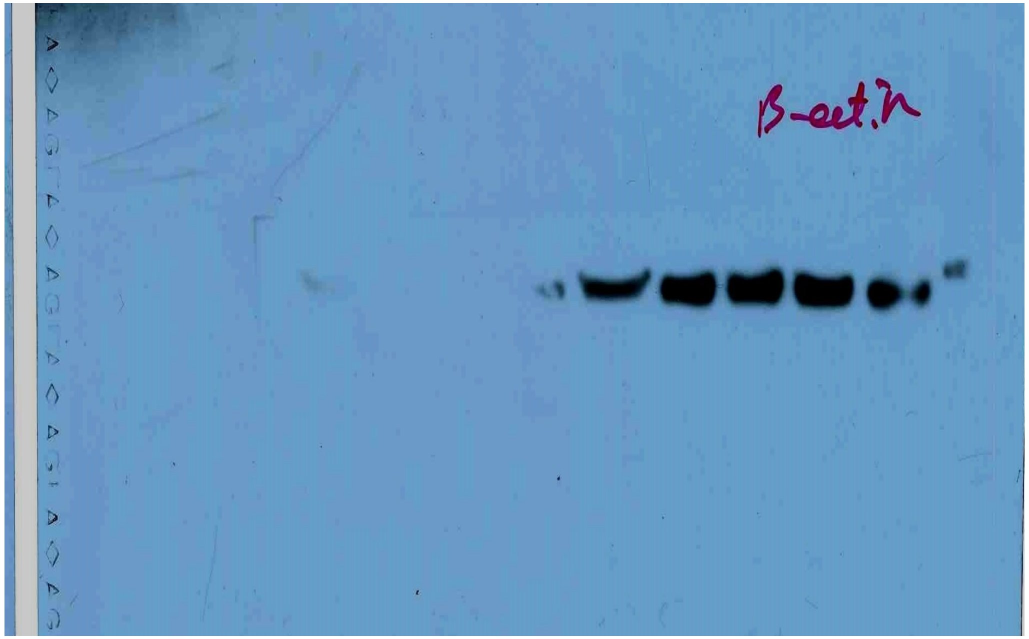

B-Actin of C2C12

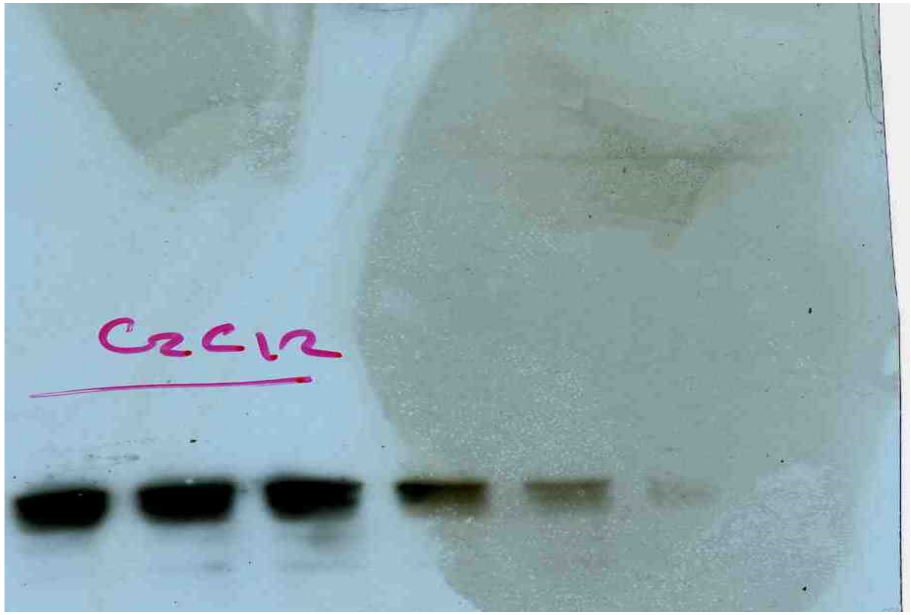

Supplement: S1 File — Each figure was named according to the related figure in the text. (PDF) [file pone.0225912.s003.pdf]
