# Supplementary material for: L-lysine protects C2C12 myotubes and 3T3-L1 adipocytes against high glucose damages and stresses
Source: PLoS One. 2019 Dec 19;14(12):e0225912. doi: 10.1371/journal.pone.0225912 (PMC6922410; doi:10.1371/journal.pone.0225912)
Supplement: Attachment — Submitted filename: Answer to Reviewer 98-08-04.docx [file pone.0225912.s005.docx]

Reviewer #1: This study investigated the effect of Lys on intracellular signaling pathways in the C2C12 myotubes and 3T3-L1 adipocyte. And found that Lys prevents the induced HG-cell death in both C2C12 myotubes and 3T3-L1 adipocytes through the reduction of oxidative stress and regulation of the UPR. I have some specific comments for the authors to consider in revising their manuscript.

1.”These figures indicate a significant decrease (p= 0.000) in the cell viability due to the HG treatment in comparison with the control cells.” and “Despite the improvement effect of Lys, the viability of the cells treated with Lys+HG was significantly (p= 0.000) lower than control cells, in both cell lines.” Which is” the control cells”, not explicitly stated in the manuscript. Figure 2 didn't show which were control cells.

Thank you very much for your kind consideration and very useful comments. We included the explanation about the control in the legend of Figure. 1. Actually, time zero (0) was considered as control with no treatment.

2."Figures 3A and 3B show the percentages of C2C12 and 3T3-L1 cells, respectively, at each quarter." However, A and B are not marked in Figure 3.

You are right. I am so sorry. The labels were added to Figure 3.

3.In Figures 4 and 5, only showed the p-value of the difference between the time points, but the p-value of the difference between the groups is not labeled.

4.In Figures 6BC and 7BC, only showed the p-value of the difference between the time points, but the p-value of the difference between the groups is not labeled.

To avoid any confusion, we did not. But, as the respected reviewer suggested, Figures 4, 5, 6BC and 7BC were additionally labeled with stars; and the stars were defined in the legends.

5.Western blot data should show loading control (β-Actin) in Figures 8 and 9.

We added the loading control (β-Actin) in Figures 8 and 9.

6.In Figures 8A, molecular weight of LC3-I/LC3-II wrong labeled.

Thank you very much. It was corrected.

7.In Figures 8 and 9, only showed the p-value of the difference between the time points, but the p-value of the difference between the groups is not labeled.

As reviewer suggested, Figures 8 and 9 were additionally labeled with stars; and the stars were defined in the legends.

8.Image and digital resolutions are unclear.

They were clarified.

9.The authors should double-check grammatical and punctuation.

Thanks for your suggestions. The grammatical and punctuation mistakes in the manuscript have been carefully checked and corrected.
